# Oxidative Stress in Cerebrospinal Fluid During Treatment in Childhood Acute Lymphoblastic Leukemia

**DOI:** 10.7759/cureus.15997

**Published:** 2021-06-28

**Authors:** Pooja Dewan, Preety Chaudhary, Sunil Gomber, Rafat S Ahmed, Mrinalini Kotru

**Affiliations:** 1 Pediatrics, University College of Medical Sciences, Delhi, IND; 2 Biochemistry, University College of Medical Sciences, Delhi, IND; 3 Pathology, University College of Medical Sciences, Delhi, IND

**Keywords:** child, leukemia, oxidative stress, reactive oxygen species, neurotoxicity

## Abstract

Introduction

Central nervous system (CNS) treatment using intrathecal chemotherapy and cranial radiation to enable long-term disease-free survival from childhood acute lymphoblastic leukemia (ALL) comes at the cost of neurotoxic side effects and long-term sequelae. We investigated oxidative stress as a possible mechanism of chemotherapy-induced neurotoxicity in children with ALL.

Materials and methods

In this case-control study, we estimated the cerebrospinal fluid (CSF) levels of 8-hydroxy-deoxyguanosine (8-OH-dG), a DNA damage product, in children with B-cell ALL and control children. CSF samples were collected at diagnosis, at end of Induction 1, Induction 2, and Induction 2A - consolidation phase. CSF 8-OH-dG levels were compared in children with and without neurotoxicity.

Results

Children with ALL (n=23) at diagnosis had significantly higher median (interquartile range, IQR) CSF 8-OH-dG levels (ng/mL) compared to controls (n=19) [1.97 (1.59-2.56) Vs 0.65 (0.59-0.82), P<0.001]. CSF 8-OH-dG levels at the end of four weeks, eight weeks, and 16 weeks of chemotherapy were [3.96 (2.85-5.44) ng/mL], 1.00 (0.89-1.09), and 3.73 (2.80-4.39) ng/mL, respectively. Out of 23 children with ALL, 12 developed neurotoxicity; the CSF levels of 8-OH-dG in them were only marginally higher compared to those who did not develop neurotoxicity. The CSF 8-OH-dG levels did not show a significant correlation with the number of doses of methotrexate or vincristine received.

Conclusion

Chemotherapy increases the CNS oxidative stress as measured by CSF 8-OH-dG levels, with the levels being proportional to the intensity of chemotherapy. Children with neurotoxicity had only marginally higher CSF 8-OH-dG levels as compared to children without neurotoxicity.

## Introduction

Acute lymphoblastic leukemia (ALL) is the most common malignant disorder among children with a peak incidence among one to five-year-old children. The central nervous system (CNS) is an important sanctuary site for leukemia cells. Therefore, CNS treatment and prophylaxis is an important component of ALL therapy, which includes the administration of intrathecal chemotherapy with drugs like methotrexate (MTX), cytosine arabinoside, and hydrocortisone, at repeated intervals in addition to cranial irradiation.

While CNS treatment has improved survival in children with ALL, it comes at the cost of the toxic effects of treatment, which may be acute, subacute, or chronic [1]. These include chemotherapeutic side effects like peripheral and cranial neuropathy, myelopathy, aseptic meningitis, and seizures as well as long-term sequelae like impaired neurocognitive abilities and neurobehavioral disturbances in toddlers and young children [2]. These effects have been associated with concomitant white matter changes seen in neuroimaging [3] and impaired nerve conduction velocity and slowing of electroencephalogram [4]. The mechanism behind such injury as well as the incidence of these brain changes is still not completely understood; impaired perfusion [5], metabolic derangements of the brain [6], and direct toxic effects on microglia [7] have been purported as likely mechanisms. The generation of reactive oxygen species (ROS) and its contribution to the neurotoxic effects of chemoradiation is also being explored [8]

Children undergoing treatment for ALL receive multiagent chemotherapy, many of these drugs like cytosine arabinoside [9], vincristine [10], and MTX [11] have been shown to induce free radical injury. Some studies have demonstrated the increased CNS oxidative stress due to chemotherapy [12-15] and shown its association with neurological sequelae [16-17].

In this case-control study, we assessed the incidence of acute and subacute neurological side effects of chemotherapy in relation to cerebrospinal fluid (CSF) levels of 8-hydroxy-deoxyguanosine (8-OH-dG), a marker of oxidative DNA damage, in children with ALL. We also measured the 8-OH-dG levels in CSF during different phases of treatment in children with ALL and as compared to controls.

We hypothesized that CSF 8-OH-dG levels are a marker of chemotherapy-induced oxidative stress and their levels are highest following phases of intensive chemotherapy and that higher CSF 8-OH-dG levels are associated with increased chemotherapy-related neurotoxicity.

## Materials and methods

This pilot study was conducted in the division of pediatric hematology-oncology of a tertiary hospital in Delhi, India, between February 2018 and April 2019. We included children (≤12 years) with newly diagnosed B-cell acute lymphoblastic leukemia in the study. Children with CNS involvement at diagnosis of ALL, and any child receiving anti-oxidants or multivitamin supplements in the preceding four weeks were excluded. Diagnosis of ALL was established by morphological examination of the bone marrow along with immunophenotyping in accordance with established WHO criteria [18]. The control group included children presenting with complex febrile seizures or seizures triggered by fever, in whom a diagnostic lumbar puncture was indicated to exclude meningitis; none of them had any serious illness, septicemia, or malignant disease. Informed written consent was obtained from all caregivers of participants and prior approval from the institutional ethics committee was obtained.

The children with B-cell ALL were treated with modified MCP 841 protocol [19], a protocol developed jointly by India and the National Cancer Institute (NCI), Bethesda, US, which neither contained high-dose methotrexate nor required estimation of minimal residual disease (MRD), and hence considered appropriate for lower-middle-income countries (LMIC). Chemotherapy for B-call ALL included an intensive phase over the initial 16 weeks followed by maintenance chemotherapy over the next 18 months, as described below.

Induction 1 (Week 1-4): Oral prednisolone 40 mg/m^2^ X 28d; intravenous (IV) vincristine 1.4 mg/m^2^/dose X 4 weekly doses Days 1, 8, 15, and 22; intravenous (IV) daunomycin 30 mg/m^2^/dose Days 8, 15, 29; IV L-asparaginase 6000 IU/m^2^/dose on alternate days (10 doses) Days 2-20; intrathecal (IT) methotrexate (MTX) (age-adjusted doses <2y: 8 mg, 2-3y: 10 mg and >3y: 12 mg) Days 1, 8, 15, and 28.

Induction 2 (Week 5-8): Oral mercaptopurine 75 mg/m^2^/dose Days 1-7, Days 15-21; IV cyclophosphamide 750 mg/m^2^/dose Days 1 and 15, IT MTX (age-adjusted doses) Days 1, 8, 15, and 22; prophylactic cranial radiation therapy (CRRT) in children older than three years in a total dose of 12 Grays (Gy) in 10 divided doses.

Repeat Induction 1 (Week 9-12): Oral prednisolone 40 mg/m^2^ X 28d; IV vincristine 1.4 mg/m^2^/dose X 4 weekly doses Days 1, 8, 15, and 22; IV daunomycin 30 mg/m^2^/dose day 8, 15, 29; IV L-asparaginase 6000 IU/m^2^/dose on alternate days (10 doses) Days 2-20; IT MTX (age adjusted doses) Days 1, 8, 15, and 28.

Induction 2A - consolidation (Week 13-16): IV Vincristine 1.4 mg/m^2^/dose Days 1 and 15, IV cyclophosphamide 750 mg/m^2^/dose Day 1, IV daunomycin 30 mg/m^2^ Day 15, IV cytosine arabinoside 2 g/m^2^/dose 12 hourly Days 1-2 and Days 15-16 (and on Days 29 and 30 for children aged <3y), oral 6-Mercaptopurine 75 mg/m^2^/day Days 1-7 and Days 15-21); IT MTX (age-adjusted doses) Days 1 and 22.

Maintenance cycle (6 cycles of 12 weeks each): IV vincristine 1.4 mg/m^2^/dose Day 1; IV daunomycin 30 mg/m^2^/dose Day 1; IV L-asparaginase 6000 IU/m^2^ on Days 1, 3, 5, and 7; oral prednisolone 40 mg/m^2^/day Days 1-7, intrathecal MTX Day 1; oral MTX 15 mg/m^2^/dose, once a week, missing every fourth week for a total of 12 weeks and beginning on Day 15; oral mercaptopurine 75 mg/m^2^/dose beginning Day 15 and given daily for three weeks out of four for a total of 12 weeks.

During treatment, a detailed clinical examination was performed to look for any evidence of chemotherapy-related toxicity. Specific neurological toxicities like neuropathic pain (jaw pain, abdominal pain), weakness in limbs, convulsions, visual or auditory disturbances, unsteady gait, or bladder or bowel disturbances were specifically enquired. A thorough clinical examination was performed on repeated encounters for any neurological deficit on follow-up. Nerve conduction velocity was performed depending upon the clinical features like weakness of limbs or detection of hyporeflexia or areflexia during the examination.

All children were closely followed up for evidence of any neurological toxicity due to chemotherapy till the initiation of the maintenance phase of chemotherapy. Chemotherapy-related toxicity was recorded, and the severity was graded as per the Common Terminology Criteria for Adverse Events (CTCAE) version 5 [20]. Serum cobalamin (vitamin B12) and folate levels were assessed by chemiluminescence assay based on competitive immunoassay technique for all children at diagnosis before initiating chemotherapy.

Estimation of CSF oxidative stress

CSF samples (2 mL) were obtained at the time of ALL diagnosis and subsequently in conjunction with therapeutic lumbar punctures for the administration of IT MTX at the completion of Induction 1 (end of Week 4), at the completion of Induction 2 (end of Week 8), and at the completion of Induction 2A - consolidation (end of Week 16). CSF samples were placed on ice immediately after collection and centrifuged for 15 min at 3000 rpm to remove any cellular debris. The CSF sample was stored at -20°C for analysis at a later date. The CSF sample was thawed and 8-OH-dG was estimated in CSF by sandwich enzyme-linked immunosorbent assay (ELISA)-based kit (Stressmarq Biosciences, Canada).

Statistical analysis

The analysis was done using Statistical Package for the Social Sciences (SPSS) software version 26 (IBM Corp., Armonk, NY). Baseline demographic characteristics were compared between the children with B-cell ALL and control groups. The chi-square test was used for comparing categorical variables and the Mann Whitney U test was used to compare non-categorical variables. 8-OH-dG levels were expressed as median (interquartile range) and compared between cases and controls by using the Mann Whitney U test. The sequential values of 8-OH-dG during different phases of treatment were compared using the Wilcoxon signed-ranks test. A subgroup analysis was to compare oxidative stress between children with B-ALL who developed neurotoxicity and those who did not develop neurotoxicity during the initial 16 weeks of treatment. A p-value of less than 0.05 was considered statistically significant. Spearman correlation was determined between CSF 8-OH-dG levels and anthropometric status, age, serum lactate dehydrogenase, total leucocyte count, lymphoblast percentage, serum cobalamine (vitamin B12), and serum folate levels measured at diagnosis (baseline). Spearman correlation was also determined between CSF 8-OH-dG levels and total doses of intrathecal methotrexate or IV vincristine received.

## Results

**Figure 1:**
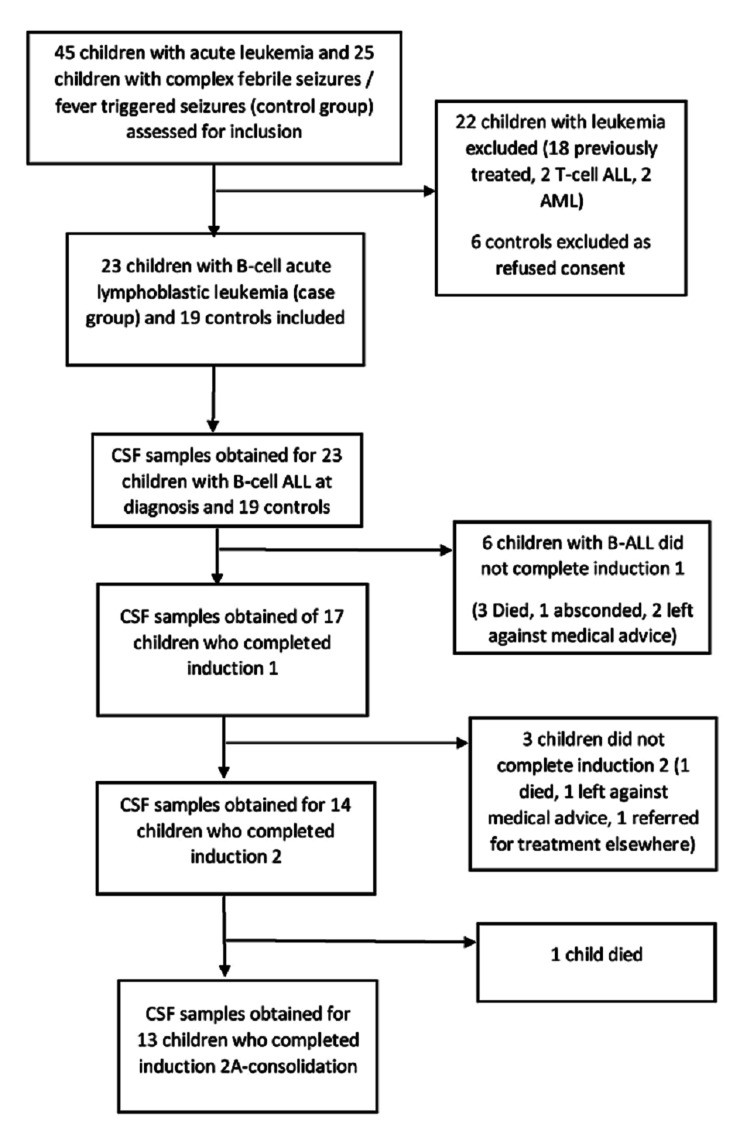
Flow chart depicting enrolment and follow-up of participants in the study

We included 23 children with newly diagnosed B-ALL and 19 controls in this study. Out of 23 children with B-ALL, 17 completed Induction 1 (initial 4 weeks therapy), 14 completed Induction 2, and 13 completed Induction 2A - consolidation. Figure 1 depicts the flow of participants in the study.

Table 1 depicts the demographic features of cases and controls. The height, weight, and body mass index (BMI) distribution was similar for children in both groups. The most common symptoms seen in children with ALL were: splenohepatomegaly (91%), pallor (91%), bleeding (83%), lymphadenopathy (83%), fever (78%), bone pains (39%), abdominal pain (26%), and weight loss (4%). The most common symptoms in the control group were fever (100%), vomiting (52%), cough/coryza (39%), and headache (8%).

**Table 1:** Demographic and Laboratory Characteristics of Children with B-Acute Lymphoblastic Leukemia (B-ALL) and Healthy Controls CSF cerebrospinal fluid; 8-OH-dG 8-hydroxy-deoxyguanosine; Values expressed as median (interquartile range)

Parameter	Children with B-ALL	Controls	P value
Age (months)	66 (36-122)	72 (48-108)	0.53
Male gender (n, %)	13, 56.5%	12, 63.2%	0.66
Weight for age Z score	-1.1 (-1.5 to 1.2)	-1.1 (-1.2 to 1.3)	0.90
Height for age Z score	1.1 (-1.2 to 1.3)	-1.2 (1.1 to 1.2)	0.27
Body mass index Z score	1.2 (-1.1 to 1.2)	-1.2 (-1.1 to 1.2)	0.11
Hemoglobin (g/dL)	6.5 (6.6-7.2)	10.1 (9.8-12)	<0.001
Total leukocyte count (cells X 10^9^/L)	18 (7.6 – 29)	6.4 (4.2-10.9)	0.013
Platelet count (cells X 10^9^/L)	29 (18.9-44)	281 (210-380)	<0.001
Serum cobalamine (pg/mL)	201 (188-258)	345 (210-456)	0.06
Serum folate (ng/mL)	5.8 (4.6-6.8)	4 (2.1-4.3)	<0.001
CSF 8-OH-dG at day 0 (ng/mL)	1.97 (1.59-2.56)	0.65 (0.59-0.82)	<0.001
CSF 8-OH-dG at week 4 (ng/mL)	3.96 (2.85-5.44)	-	-
CSF 8-OH-dG at week 8 (ng/mL)	1.00 (0.89-1.09)	-	-
CSF 8-OH-dG at week 16 (ng/mL)	3.73 (2.80-4.39)	-	-

**Figure 2:**
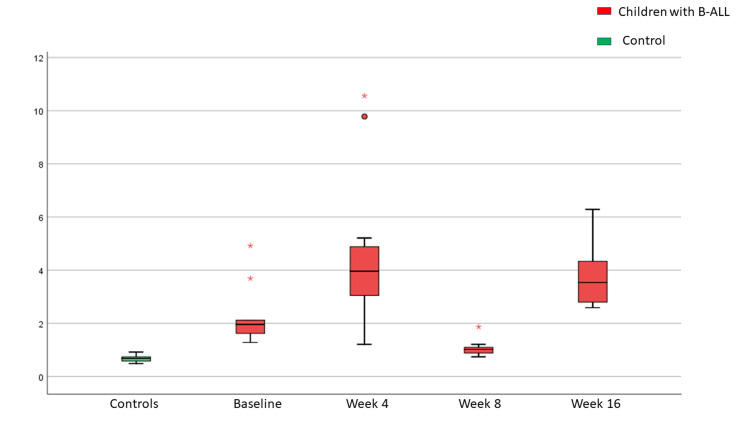
Cerebrospinal fluid oxidative stress as measured by 8-hydroxy-deoxyguanosine (8-OH-dG) levels (ng/mL) during chemotherapy in children with acute lymphoblastic leukemia and controls

In children with ALL, the CSF oxidative stress as measured by 8-OH-dG levels was highest at the end of Induction 1 (week 4) and was least after Induction 2 (week 8) and then rose again following Induction 2A - consolidation phase (week 16) as seen in Table 1. The sequential change in 8-OH-dG levels during different phases of the treatment showed a significant difference between baseline and end of Induction 1 (P<0.001), end of Induction 1, and end of Induction 2 (P<0.001), end of Induction 2, and end of Induction 2A - consolidation (P<0.001), and baseline and end of Induction 2A - consolidation (P<0.001). The CSF oxidative stress in children with ALL remained significantly higher than that seen in the control group at all time points during chemotherapy (P<0.001), as shown in Figure 2.

Significant correlation was observed between CSF oxidative stress (8-OH-dG levels) and body mass index for age Z score (BMIZ) (r=0.51, P=0.01) but not with age (r=0.25, P=0.26), weight for age Z score (WAZ) (r=0.33, P=0.12), height for age Z score (HAZ) (r=0.40, P=0.05), hemoglobin level (r=o.14, P=0.51), total leucocyte count (r=0.14, P=0.12), platelet count (r=0.14, P=0.51), serum cobalamine levels (r=0.26, P=0.23), serum folate (-0.15, P=0.50), and serum lactate dehydrogenase levels (r=0.20, P=0.36). No correlation was seen between CSF 8-OH-dG levels and number of doses of intrathecal methotrexate received (r=-0.10; P=0.51) or number of doses of vincristine received (r=0.29, P=0.05).

During chemotherapy, neurological side effects were seen in 12 children with B-ALL (Table 2). Five of these children complained of weakness in lower limbs and difficulty in walking and rising from bed during the third or fourth week of induction therapy; clinical examination confirmed motor weakness in lower limbs with a power of ≤ 3/5 in bilateral extensors and flexors at knee and hip joint with diminished deep tendon reflexes in lower limbs. Nerve conduction velocity (NCV) studies confirmed axonal neuropathy in all five of them. The weakness improved in all five subjects during follow-up over the next four to five weeks, and no child had any residual motor deficit. Seven children complained of jaw pain and difficulty in chewing during Induction 1 phase of chemotherapy; the pain was attributed to vincristine-induced sensory neuropathy, as serum cobalamine levels were normal and the pain was temporally associated with vincristine administration; the neuropathic pain relieved without intervention in six children while one child needed pharmacotherapy using carbamazepine. Two children each complained of ataxia and seizures during chemotherapy. MRI in the children with seizures revealed white matter changes and the seizures were controlled with anti-epileptic drugs. Neuroimaging in the children with ataxia was unremarkable.

**Table 2:** Profile of chemotherapy-related neurotoxicity in children with ALL (n=23) Values expressed as n (%) ALL: acute lymphoblastic leukemia

Symptom	Common Toxicity Criteria Grading		
	Grade 0	Grades 1,2	Grades 3,4
Motor neuropathy (weakness in limbs)	18 (78.3%)	4 (17.4%)	1 (4.3%)
Sensory neuropathy (jaw pain, abdominal pain)	16 (69.6%)	7 (30.4%)	0
Ataxia	21 (91.3%)	2 (8.6%)	0
Seizures	21 (91.3%)	0	2 (8.6%)

Table 3 depicts the comparative demographic and laboratory features of children with and without neurotoxicity. The CSF oxidative stress was statistically comparable between children with and without neurotoxicity at all four time points of assessment (baseline, end of weeks 4, 8, and 16).

**Table 3:** Comparative demographic and laboratory parameters in children with and without chemotherapy-related neurotoxicity Values expressed as *median (interquartile range) or #number (%) CSF: cerebrospinal fluid; 8-OH-dG: 8-hydroxy-deoxyguanosine

Parameters	With chemotherapy-related neurotoxicity	Without chemotherapy-related neurotoxicity	P-value
Age (months)*	61 (34.5-129.5)	60 (36-108)	0.93
Male sex#	8 (66.7%)	5 (45.6%)	0.30
Weight for age Z score (WAZ)*	-1.1 (-1.97 to 1.2)	-1.1 (-1.4 to 1.2)	0.83
Height for age Z score (HAZ)*	1.2 (-1.35 to 1.27)	1.1 (-1.1 to 1.2)	0.92
Body Mass Index for age Z score (BMIZ)*	1.2 (-0.7 to 1.27)	1.2 (-1.1 to 1.2)	0.65
Serum cobalamine (pg/mL)*	197.5 (161.25 to 266.5)	201 (188-258)	0.52
Serum folate (ng/mL)*	5.8 (4.72 to 6.9)	5.8 (4.1 to 6.8)	0.83
CSF 8-OH-dG at day 0*	2.0 (1.5 to 2.6)	1.97 (1.59 to 3.39)	1
CSF 8-OH-dG at week 4*	4.2 (2.9 to 3.2)	3.9 (2.6 to 5.7)	0.73
CSF 8-OH-dG at week 8*	1.0 (1.0 (0.88 to 1.1)	1.0 (0.89 to 1.07)	0.84
CSF 8-OH-dG at week 16*	3.79 (2.67 to 4.99)	3.54 (3.21 to 3.9)	0.94

## Discussion

With the use of CNS-directed chemotherapy and radiation, the five-year survival from childhood ALL has increased considerably. In India, the overall five-year survival in children with ALL ranges from 65%-85% [21-22], although up to 85% of survivors experience CNS treatment-related neurological problems [23-24]. Despite the high incidence of neurological sequelae, little is known about the mechanisms of neurological damage. We investigated oxidative stress, measured by the CSF levels of 8-OH-dG, a DNA damage product, during the different phases of chemotherapy in children with ALL and studied its relationship with neurological side effects of chemotherapy.

There is ample experimental evidence to suggest that chemotherapeutic agents act by oxidative damage to lipids of cellular membranes, proteins, and DNA in tumor cells [25]. It has also been shown that flavoproteins transfer electrons from nicotinamide adenine dinucleotide hydrogen (NADH) and flavin adenine dinucleotide hydrogen (FADH) to chemotherapeutic drugs like doxorubicin, a drug commonly used in the treatment of hematological malignancies including leukemia. Further, reduction of oxygen to superoxide regenerates the original doxorubicin; the generation of ROS in this process can lead to oxidative damage to tissues [26]. 8-hydroxy-2-deoxyguanosine (8-OH-dG) or 8-oxo-7,8-dihydro-2-deoxyguanosine (8-oxo-dG) are the two byproducts of free radical-induced oxidative damage to cellular DNA. Previously, 8-OH-dG and 8-oxo-dG have been studied as a biomarker of free radical injury in blood, bone marrow [27-28], and urine [29] of children with ALL receiving chemotherapy, although it has not been estimated in CSF. CSF oxidative stress has been ascertained in children with ALL using products of lipid peroxidation like oxidized components of phosphatidylcholine (PC) and phosphatidylinositol (PI), the most prevalent phospholipid in CNS cellular membranes [12,15] and isoprostane [13].

As hypothesized by us, we found that 8-OH-dG levels increased significantly after the initiation of chemotherapy and peaked at the end of four weeks with the completion of aggressive induction chemotherapy. We noticed that the oxidative stress declined over the next one month despite cranial radiation; probably due to much less intensive chemotherapy in the second month of treatment in our protocol and the use of only prophylactic doses of cranial radiation therapy. Subsequently, the CSF levels of 8-OH-dG increased significantly at the end of Induction 2A - consolidation, a fairly intensive treatment phase involving the administration of high-dose IV cytosine arabinoside and IV cyclophosphamide. Similar trends of highest oxidative stress in children receiving treatment for ALL at diagnosis and at the end of the consolidation phase were reported by others [12,14,16]. Our findings emphasize that systemically administered chemotherapy drugs, as well as IT methotrexate, increase oxidative stress in the brain.

Nearly 50% of our children developed neurotoxicity although no significant difference was seen in CSF levels of 8-OH-dG in children with and without neurological complications. Unlike previous studies wherein a direct correlation was seen between levels of oxidized PI and total intravenous methotrexate received [14], we did not find a direct correlation between levels of 8-OH-dG and total doses of IT MTX or IV vincristine. It is possible that products of lipid peroxidation are more sensitive markers of CNS oxidative stress as compared to DNA damage products like 8-OH-dG. A previous study by Faure et al. [30] observed that the DNA damage product 5-hydroxymethyluracil in urine proved to be a better biomarker than 8-oxo-7,8-dihydroguanosine to assess doxorubicin-induced DNA damage; the role of different biomarkers to determine oxidative stress needs to be evaluated. Also, unlike Moore et al. [14], the chemotherapeutic protocol followed by us did not use IV MTX, which has been shown to be significantly more toxic. Like Protas et al. [15], we also could not find any correlation between 8-OH-dG levels in CSF and tumor burden as measured by total lymphoblasts count (percentage), total leucocyte count, and serum lactate dehydrogenase levels. Whether the relationship between raised 8-OH-dG levels in CSF and neurotoxicity is co-incidental needs to be explored in future studies, as well as the clinical implications of this association, if any.

Strengths of our study include the presence of a control group, unlike the previous studies [12-15]. Our findings of significantly greater 8-OH-dG CSF levels in children with ALL compared to controls indicate that ALL itself leads to CNS oxidative stress even in the absence of CNS involvement. Previously, another study [29] demonstrated that levels of urinary 8-OH-dG were higher in the newly diagnosed children with ALL (n=8) compared with the control group (n=8), although the difference was not statistically significant. The lack of significance observed by them may have been due to the small sample size. Also, the distribution of this DNA damage product across different body fluids may have been different.

Another strength of our study includes the homogenous population of children with B-cell ALL and exclusion of children with CNS leukemia or relapsed ALL. The limitations of our study include a small sample size with the absence of long-term follow-up to evaluate neurological sequelae in the survivors.

## Conclusions

Our study provides preliminary evidence that chemotherapy increases the CNS oxidative stress as measured by CSF 8-OH-dG levels, with the levels being proportional to the intensity of chemotherapy. Children with neurotoxicity had only marginally higher CSF 8-OH-dG levels as compared to children without neurotoxicity. Longitudinal studies of ALL survivors are needed to determine whether the CNS oxidative stress persists over time and to evaluate the same in relation to neurological sequelae.
